# Rupture of the Uterus

**Published:** 1901-07-20

**Authors:** 


### RUPTURE OF THE UTERUS.

AT the last meeting of the Obstetrical Society, a paper was read by Dr. Preston Maxwell on "Spontaneous Rupture of the Uterus in Placenta Prævia." He related three cases in which this accident had occurred. The chief interest in the discussion centered in the treatment employed in the two cases which recovered. The first woman died undelivered within a few minutes of the rupture. In the other two, although there had been dangerous hæmorrhage, recovery took place, and both of them were packed with antiseptic gauze. Dr. F. H. Champneys said that the plugging of the rent with gauze was the most successful treatment in cases in which the fœtus and placenta had not escaped into the peritoneal cavity. Dr. Herbert Spencer said that he had called attention to this method of treatment in a paper which he read before the society last year, giving notes of four cases which had been successfully treated by gauze packing. His previous experience had been that in every case of rupture of the uterus (about eight in all) the patient had died. At the present time he said it was usual to recommend abdominal section for complete rupture of the uterus, but that operation, especially if followed by hysterectomy, was generally too severe a shock for a patient suffering from a rupture of the uterus, and if those who had had experience of that accident would publish all their cases, as he had done, he had no doubt that the results of hysterectomy would compare very unfavourably with those given by gauze packing. The President (Dr. Peter Horrocks) also spoke in favour of gauze packing, but he pointed out that its success was largely dependent upon the fact that the parts had not been rendered septic by the surgeon's hands.

